# QSAR models reveal new EPAC-selective allosteric modulators†

**DOI:** 10.1039/d2cb00106c

**Published:** 2022-08-03

**Authors:** Hebatallah Mohamed, Hongzhao Shao, Madoka Akimoto, Patrick Darveau, Marc R. MacKinnon, Jakob Magolan, Giuseppe Melacini

**Affiliations:** Department of Chemistry and Chemical Biology, McMaster University, Hamilton Ontario L8S 4L8 Canada melacin@mcmaster.ca; Department of Biochemistry and Biomedical Sciences, McMaster University, Hamilton Ontario L8S 4L8 Canada

## Abstract

Exchange proteins directly activated by cAMP (EPAC) are guanine nucleotide exchange factors for the small GTPases, Rap1 and Rap2. They regulate several physiological functions and mitigation of their activity has been suggested as a possible treatment for multiple diseases such as cardiomyopathy, diabetes, chronic pain, and cancer. Several EPAC-specific modulators have been developed, however studies that quantify their structure–activity relationships are still lacking. Here we propose a quantitative structure–activity relationship (QSAR) model for a series of EPAC-specific compounds. The model demonstrated high reproducibility and predictivity and the predictive ability of the model was tested against a series of compounds that were unknown to the model. The compound with the highest predicted affinity was validated experimentally through fluorescence-based competition assays and NMR experiments revealed its mode of binding and mechanism of action as a partial agonist. The proposed QSAR model can, therefore, serve as an effective screening tool to identify promising EPAC-selective drug leads with enhanced potency.

## Abstract 2

QSAR models of EPAC-specific allosteric ligands predict the affinity of a promising analogue.

## Graphical abstract

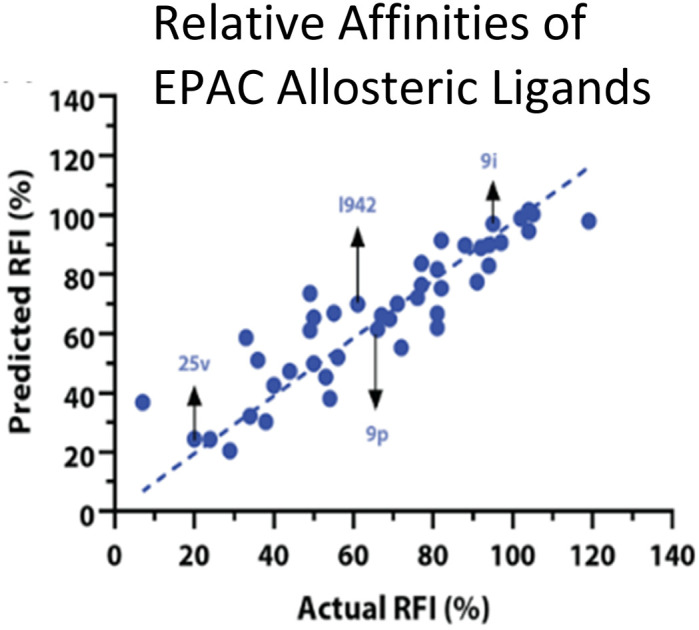

## Introduction

Exchange proteins directly activated by cyclic adenosine monophosphate (cAMP), commonly referred to as ‘EPAC’, are guanine nucleotide exchange factors (GEFs) that mediate guanosine diphosphate (GDP) to guanosine triphosphate (GTP) exchange on small GTPases, Rap1 and Rap2.^1,2^ Two main isoforms of EPAC have been discovered, EPAC1 and EPAC2, and though they share structural similarity, their biological function and tissue distribution are different.^3^ Modulation of EPAC has been proposed to be a gateway for treatment of several diseases, including several cardiovascular disorders.^4^ In particular, a recently discovered EPAC-selective partial agonist, known as I942^5^ has been shown, through Rap1 GEF assays, to bind competitively to EPAC, and its activity has been proposed as a potential means to suppress pro-inflammatory pathways associated with serious cardiovascular diseases.^6^

**Fig. 1:**
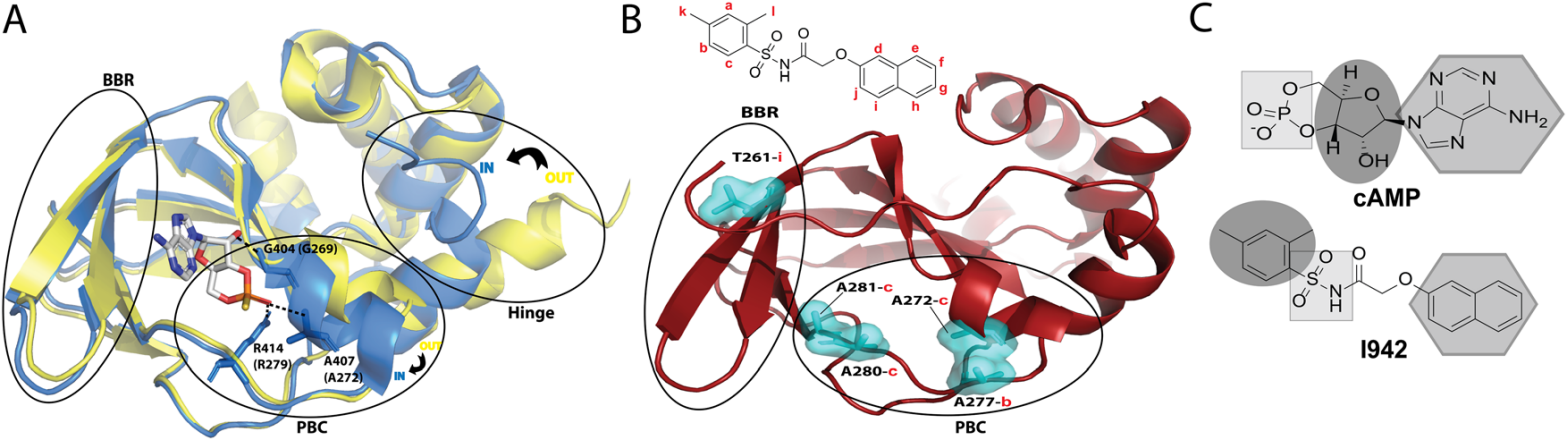
(A) The cAMP-bound EPAC2-CNBD (holo) state (blue, PDB 3CF6)^10^ is overlayed on the unbound (apo) state (yellow, PDB 1O7F).^9^ The phosphate binding cassette (PBC), the base binding region (BBR) and the hinge region are marked by black circles. The PBC and the hinge are in an ‘out’ conformation in the apo state and adopt an ‘in’ conformation in the holo state. cAMP, illustrated in sticks, forms key interactions between its phosphate group and residues in the PBC whereby equivalent residues in EPAC1 are written in brackets. The base of the nucleotide is oriented towards the BBR. (B) Map of residues (cyan surfaces) in the EPAC1-CNBD that showed NOE peaks to I942 protons.^8^ NOE peaks to protons in the phenyl group of I942 (2 and 3) originated from residues at the PBC, whereas the NOE peak towards the naphthalene proton (9) originated from the BBR residue, T261. (C) A scheme for the cAMP *vs.* I942 mimicry whereby regions of the molecular structures that are highlighted by the same shape are proposed to interact with similar regions of EPAC1-CNBD.

I942 functions by binding to the cyclic nucleotide binding domain (CNBD) of EPAC, which serves as the critical allosteric element^7^ regulating EPAC activation. Recent NMR studies on how I942 interacts with the EPAC1 CNBD have provided initial insight on the I942 mechanism of action.^8^ The unbound EPAC form (apo) exhibits a dominant inactive state where the phosphate-binding cassette (PBC), which is the region that binds the cAMP phosphate group, is in the ‘out’ conformation (Fig. 1A). The C-terminal helix of the CNBD, α6 helix, commonly called the hinge region as it controls the relative rotation of the regulatory and catalytic regions, also exhibits an ‘out’ conformation (Fig. 1A). Upon cAMP binding the PBC shifts to the ‘in’ conformation and the hinge, consequently, moves to the ‘in’ conformation since it is allosterically coupled to the PBC.^9–16^

A binding interface was proposed for I942 and the EPAC1-CNBD based on measurements of intermolecular nuclear Overhauser effects (NOEs).^8,17,18^ Such NOEs (Fig. 1B) are between the PBC residues and the I942 protons in the dimethylbenzene and between the base binding region (BBR) residues and the I942 naphthalene moiety. Based on those NOEs as well as chemical shift perturbation analyses, I942 was proposed to mimic cAMP (Fig. 1C), whereby the adenine base of cAMP or the naphthalene group in I942 interact with the BBR, whereas the cAMP's ribose ring or the I942 dimethylbenzene group interact with the PBC region.^8^ Moreover, the I942 binding induces a partial shift in the PBC and hinge regions and results in a partial ‘in-to-out’ conformational transition in those two regions. The observed effect is more dominantly seen in the hinge region, and together with the chemical shift covariance analysis (CHESCA) results that reported weakened allosteric couplings, a mixed intermediate state was proposed for the I942: EPAC1-CNBD complex, whereby the PBC is in the ‘in’ conformation and the hinge is in the ‘out’.^8^

Since I942 showed promising EPAC1-selective activity, Wang *et al.*^19^ evaluated several I942 analogs for their EPAC binding affinities. Here, we show how it is possible to develop a quantitative structure–activity relationship (QSAR) based on such affinities and how such QSAR can be used to design new I942 analogs with improved affinity for EPAC1. QSAR models aim at establishing a statistically significant correlation between a target property, such as the inhibition of an enzyme function, and molecular descriptors of ligands.^20,21^ The concept of QSAR modeling was originally introduced in 1964 by Hansch and Fujita.^22^ Since then, it has been extensively applied in the computer-aided drug discovery process, yet rarely to CNBDs.^23^

QSAR modeling relies on the notion that compounds which share structural similarity often display comparable biological activities, and this is known as the similarity-property principle (SPP).^21^ The SPP proposes that slight structural modifications of a compound correspond to slight variations in a biological property, such as potency, of that compound which, in turn, creates the foundation for linear relations that QSAR models aim to generate. From these linear relations, QSAR models can then be utilized for potency predictions of new ligands with a conserved scaffold and varying substituents.

Although several notable advancements in QSAR methods have been developed in the last three decades, only a limited number of QSAR studies are currently available for proteins involved in the cAMP-mediated signaling cascade and none for EPAC.^24–28^ Here, we report a reliable QSAR model generated using the aforementioned series of I942 analogues and their respective affinity measurements towards the EPAC1-CNBD. We then validated the QSAR model and used it to predict affinities for a series of I942 analogues that were ‘unknown’ to the model. The affinity for the most promising candidate, known as MLGM-2013, as predicted by our validated QSAR model, was confirmed through fluorescence competition assays. In addition, we investigated the mechanism of action of MLGM-2013 using NMR experiments,^29^ revealing a new avenue to design I942 analogs with enhanced potency through modifications of its phenyl moiety.

## Results and discussion

### QSAR model development

**Fig. 2:**
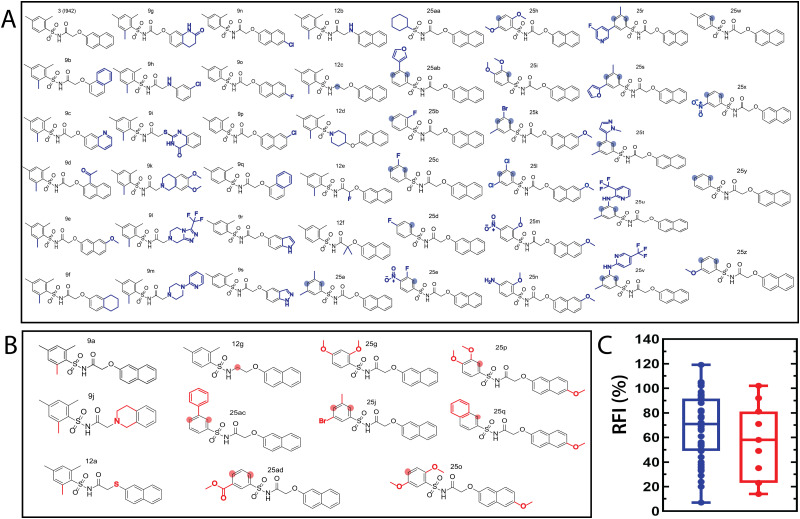
Molecular structures of the I942 analogues in the (A) training and (B) test sets. The training and test sets include 45 and 11 molecules, respectively. The modifications, relative to I942 (3), are marked by blue for the training and red for the test set. The shaded circles highlight the positions that lack substituents originally found in I942. (C) Box plot representation for the distribution of the RFI values in the training (blue) and test (red) sets.

**Fig. 3:**
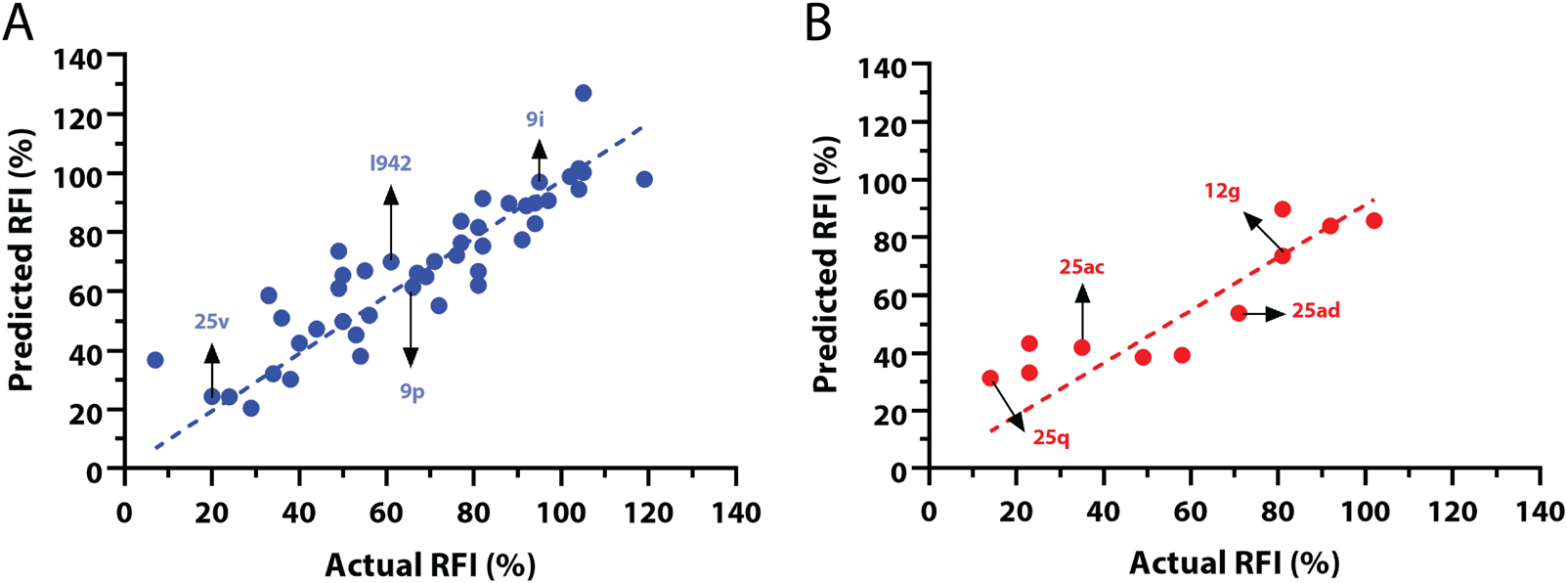
Predicted *vs.* measured relative fluorescence intensities (RFI) correlation plots, with zero intercepts, for the (A) training and (B) test sets of I942 analogues shown in Fig. 2. Representative molecules are marked with black arrows and labeled in their assigned compound names as in Fig. 2.

The QSAR models for I942 were developed according to the flowchart described in Fig. S1 (ESI†), where the partitioning of training and test sets was implemented maintaining an 80 : 20 ratio (Fig. 2A and B) and a balanced distribution of affinities, as quantified by relative fluorescence intensity (RFI) percentage values^19^ (Fig. 2C). The RFI percentages are derived from a competition-based assay^30^ where a fluorescently-tagged cAMP with high affinity towards EPAC, known as 8-(2-[7-nitro-4-benzofurazanyl] aminoethylthio) adenosine-3′,5′-cyclic monophosphate (8-NBD-cAMP), is added to the CNBD of EPAC1 in the presence of an I942 analogue. The higher the affinity of the analogue, the greater the displacement of 8-NBD-cAMP and the lower the fluorescence intensity (FI) which in turn, translates into a lower FI percentage relative to the FI of EPAC1-CNBD in presence of only 8-NBD-cAMP.^19^ QSAR models were developed for a total of 11 distinct training *vs.* test set partitions in compliance with the same criteria (Fig. S2 and Table S1, ESI†). The average values of statistical parameters describing the QSAR quality were then computed across the 11 resulting QSAR models (Table 1). One of the primary QSAR quality descriptors is the coefficient of multiple determination,^31^ referred to as *R*^2^, which reflects the overall accuracy of the RFI values predicted by the model compared to the actual measured RFI percentages. As seen from Table 1, the *R*^2^ values are high (above threshold) for both training and test sets, reflecting the ability of the model to reproduce the original data as well as to predict external data respectively. The data points in the correlation plots are also closely arranged around the line of best fit set to have a zero intercept (Fig. 3). We also computed the average statistical parameters of the 11 QSAR models without imposing a zero-intercept (Table S2 and Fig. S3, ESI†), showing slightly improved performance with the intercept set to zero (Fig. 3). Furthermore, the Y-randomization test^32^ was performed on the QSAR model from Fig. 2 and 3 to confirm that the correlation between the descriptors and the RFI values was not due to mere chance and the test was done over a 1000 iterations of perturbed Y values (the RFI values in this study). The model's robustness was measured through the Y-randomization coefficient, ^c^*R*_p^2^_, which was 0.72, and that is above the threshold value of 0.5.

**Table 1:** Parameters for the QSAR model developed for the I942 analogues^a^

	Training set^b^^c^	Test set^d^	Cross-validation (CV)	Threshold^36,37^
*R* ^2^	0.972 ± 0.008	0.929 ± 0.021	0.772 ± 0.055	*R* ^2^ > 0.600 and > 0.500 for CV
*σ*	27.69 ± 0.60	28.69 ± 2.40	—	—
RMSE	11.83 ± 1.81	19.09 ± 3.13	12.78 ± 1.50	RMSE < *σ*
*k*	0.972 ± 0.008	0.960 ± 0.090	—	0.850 ≤ *k* ≤ 1.150

a Standard deviations were computed using data from eleven different partitioning of training *vs.* test sets.

b Number of molecules in the training set was 45.

c Number of descriptors were ≤9.

d Number of molecules in the test set was 11.

Interestingly, the QSAR models obtained from the 11 distinct partitions, exhibited classes of recurring molecular descriptors in the multiple linear regressions. The shared descriptors are 2D in nature and fall in the ‘autocorrelation’ category, which essentially captures the distribution of physicochemical properties across the spatial arrangement of atoms.^33^ They describe the correlation between values of specific functions placed at intervals referred to as ‘*lag*’. The specific functions are the atomic physicochemical properties and ‘*lag*’ represents the topological distance at which these properties are distributed.^34^ In our particular model, the main physicochemical properties are (a) the intrinsic state, represented by the GATS5s, AATS5s and MATS5s descriptors, and these report on the electronegativity of the atom in its valence state, as well as (b) the Sanderson electronegativity^35^ represented by descriptors such as ATSC8e and AATS5e. It was interesting to observe consistently positive coefficients for the descriptors in the linear regression equations, which reflects a positive correlation between these descriptors and the RFI values.

### Affinity prediction of unknown compounds and experimental validation through fluorescence competition assays

**Fig. 4:**
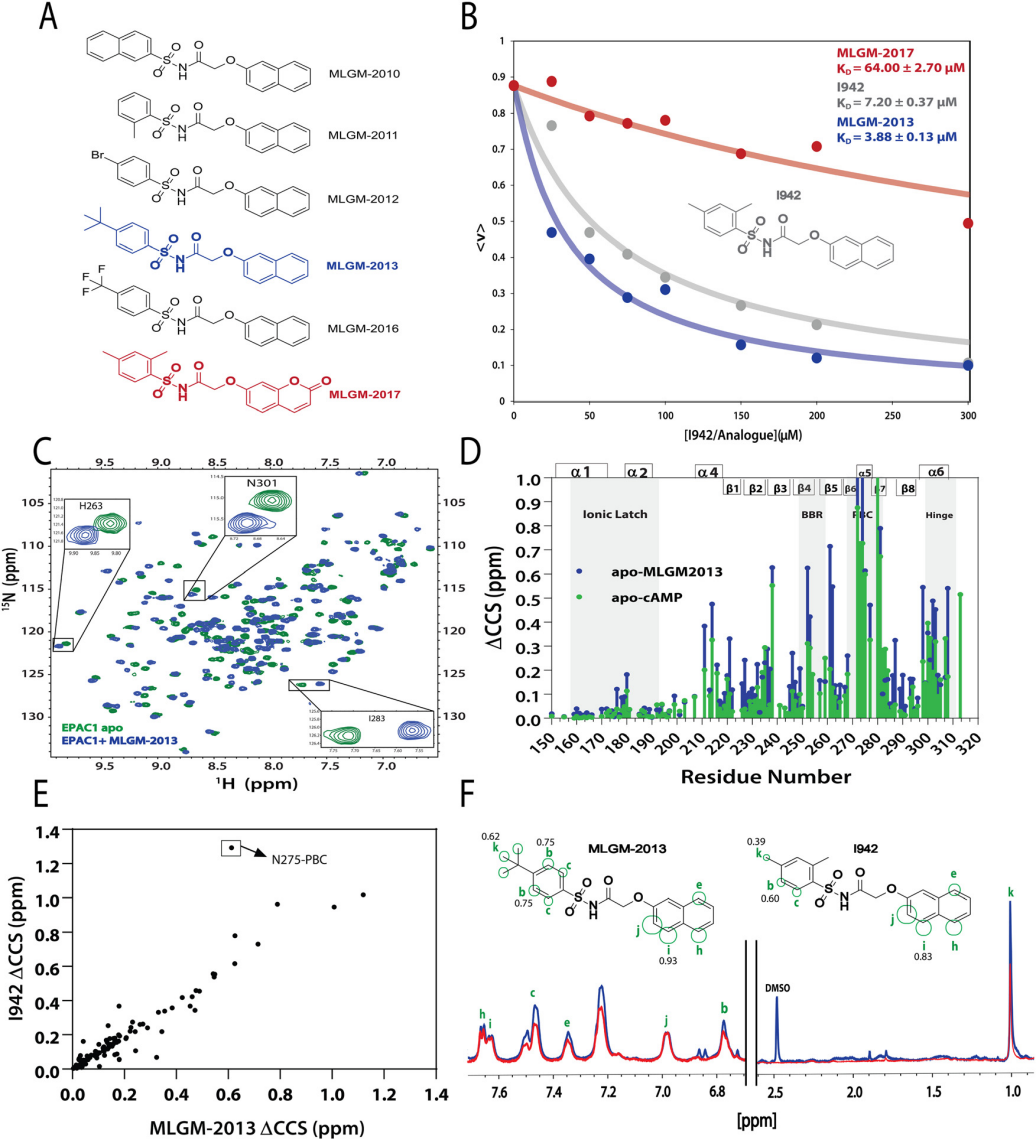
Independent validation of the QSAR model. (A) Molecular structures of new synthesized I942 analogues that are ‘unknown’ to the QSAR model. MLGM-2013, which was predicted to have the highest affinity towards EPAC1-CNBD (Table 2), is highlighted in blue and MLGM-2017, the compound with the lowest predicted affinity (Table 2) is shown in red. (B) EPAC1-CNBD binding isotherm for I942 (grey), MLGM-2013 (blue), and MLGM-2017 (red) measured through the 8-NBD-cAMP fluorescence-based competition assay. The resulting measured dissociation constants are included in the top right corner. The percentage of 8-NBD-cAMP bound to EPAC1 is represented by 〈*v*〉 on the *y*-axis. (C) The chemical shift differences between 50 μM apo EPAC1-CNBD (green) and 50 μM EPAC1-CNBD bound to 350 μM of MLGM-2013 (blue) were monitored by ^15^N-^1^HSQC spectra. (D) The compounded chemical shift variations between 50 μM apo EPAC1-CNBD and 50 μM EPAC1-CNBD bound to MLGM-2013 (350 μM), and 100 μM apo EPAC1-CNBD and 100 μM EPAC1-CNBD bound to cAMP (1 mM) are plotted as blue and green bars, respectively. The secondary structure is shown on the top of the plot in boxes and key regions are highlighted in grey. (E) Compounded chemical shift differences of 50 μM EPAC1-CNBD in the presence of MLGM-2013 (350 μM) are plotted against the compounded chemical shift differences of 50 μM EPAC1-CNBD in the presence of I942 (350 μM). (F) 1D saturation-transfer reference (STR, blue) spectrum of EPAC1-CNBD: MLGM-2013 overlayed with the scaled saturation transfer difference (STD, red) spectrum. The assigned protons are marked in green and represented as circles on the structure of MLGM-2013 where the size of the circles reflects the relative STD/STR ratios (normalized to proton j with the highest STD/STR ratio). The structure of I942 with the previously determined STD/STR ratios^8^ are shown for comparison whereby the STD/STR ratios with the most significant differences are reported near the corresponding proton.

After model validation, both internal, through the training set and cross-validation *R*^2^, and external, through the test set *R*^2^ (Fig. 2), the model with the highest test set *R*^2^ value (considering both zero and non-zero intercepts) was used to predict the RFI values of a new set of I942 analogues that were not part of either the original training or test sets (Fig. 4A). Therefore, the I942 derivatives in the new set, referred to with the ‘MLGM’ code in Fig. 4A, are essentially ‘unknown’ to our QSAR model, but they all share the same skeleton common to other I942 analogues with a sulfonamide flanked by phenyl and linked naphthyl moieties (Fig. 4A). Based on the RFI values predicted by our QSAR model for the new set of I942 analogs (Table 2), the MLGM-2013 derivative (Fig. 4A) stood out as having the lowest predicted RFI, pointing to better binding affinity for EPAC1 relative to I942. On the contrary, the MLGM-2017 derivative (Fig. 4A) was predicted to exhibit the weakest EPAC1 affinity (highest RFI value) within the new set.

**Table 2:** Predicted RFI values for a series of I942 analogues ‘unknown’ to the QSAR Model

Compound name	Predicted RFI (%)
MLGM-2013	25.87
MLGM-2010	48.73
MLGM-2016	52.32
MLGM-2011	59.36
I942	69.97
MLGM-2012	79.08
MLGM-2017	82.22

To confirm our predictions, a competition assay was preformed using the fluorescently tagged cAMP known as 8-(2-[7-nitro-4-benzofurazanyl] aminoethylthio) adenosine-3′,5′-cyclic monophosphate (8-NBD-cAMP).^30^ The displacement of 8-NBD-cAMP by a competing ligand at increasing concentrations was used to measure the dissociation constant (*K*_D_) of MLGM-2013, I942 as well as MLGM-2017, as a negative control. The assay clearly showed a significant enhancement of the binding affinity of MLGM-2013 relative to I942 (Fig. 4B), while MLGM-2017 resulted in a significantly higher *K*_D_ value compared to that of I942, as expected, further confirming the validity of our QSAR model's predictions. To gain structural insight into the enhanced affinity of MLGM-2013 and its mechanism of action, we also investigated the interactions of this I942 analog with the EPAC1 CNBD using NMR.^38,39^

### The MLGM-2013 binding mode

The binding of MLGM-2013 to the EPAC1 CNBD was monitored through ^15^N–^1^H-HSQC spectra^40^ (Fig. 4C) and the corresponding chemical shift changes (ΔCCS) are reported in Fig. 4D. Fig. 4D shows major ppm variations induced by MLGM-2013 in key CNBD regions such as the BBR, the PBC and the hinge region, quite similar to the chemical shift changes observed upon cAMP binding (Fig. 4D). The ΔCCS measured for MLGM-2013 are also quite similar to those observed for I942 (Fig. 4E), suggesting a similar binding mode, with the notable exception of N275 located in the PBC (Fig. 4E).

To further elucidate the difference in binding affinity between I942 and MLGM-2013, saturation transfer difference (STD) experiments were performed to map the binding epitopes of MLGM-2013 and assess the proximity of ligand protons to the EPAC1-CNBD (Fig. 4F).^8,41^ Interestingly, we found that the STD/STR ratios for MLGM-2013 are higher for several phenyl protons compared to I942 with the most significant increase observed for the tertiary butyl protons located at the para position of the phenyl group (Fig. 4F and Fig. S4, ESI†). As opposed to the single methyl group at that location in I942, the additional methyls of the tertiary butyl offer more extensive contacts with the protein as seen through STD/STR ratios of 0.62 *vs.* 0.39 for MLGM-2013:EPAC1-CNBD *vs.* I942:EPAC1-CNBD, respectively (Fig. 4F). Based on the N275 outlier observed in Fig. 4E, we hypothesized that the enhanced contacts of the tertiary butyl in MLGM-2013 are with the PBC of the EPAC1-CNBD.

**Fig. 5:**
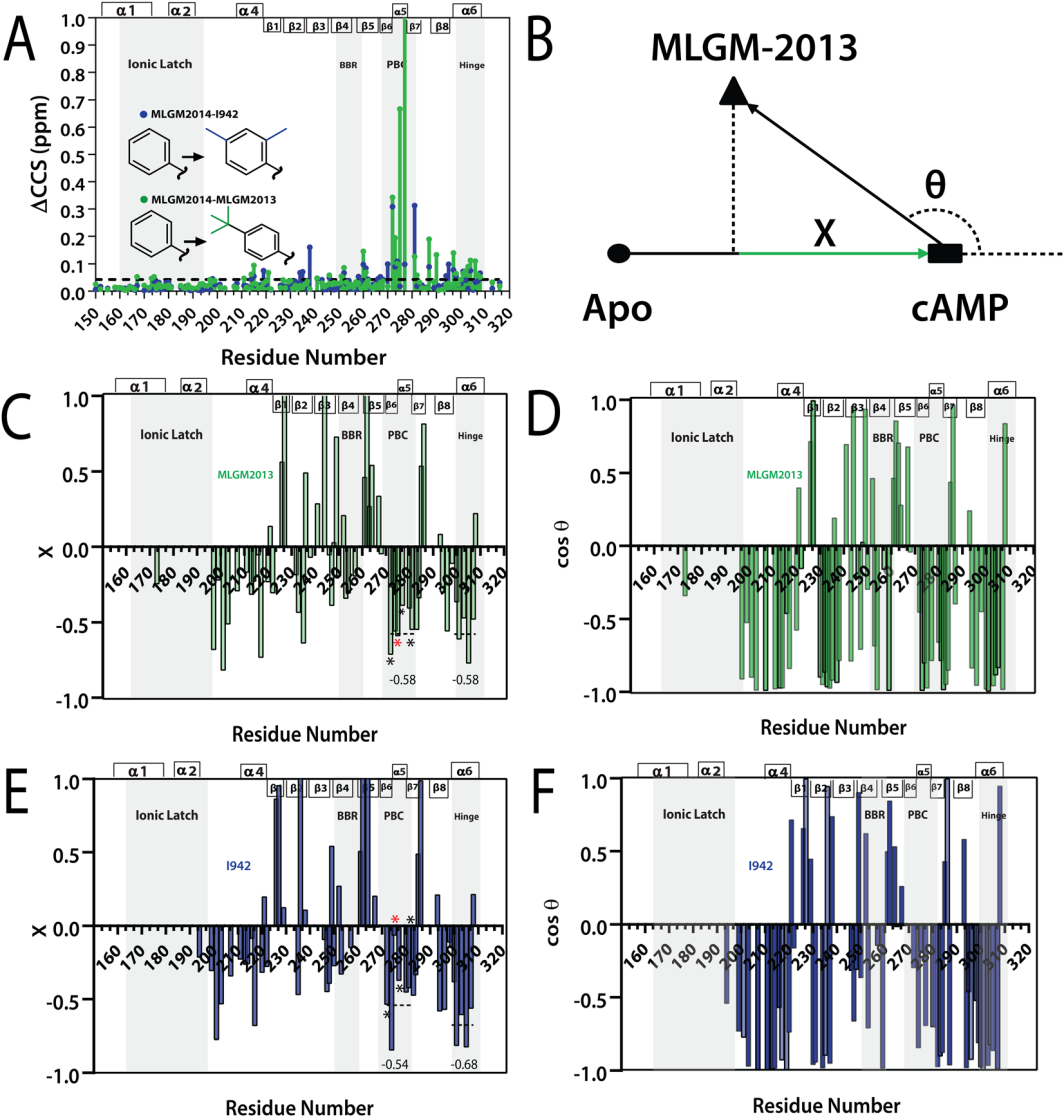
(A) Residue specific compounded chemical shift variations between MLGM-2013-bound (green) or I942-bound (blue) and MLGM-2014-bound EPAC1 CNBD. Structural differences between the ligands are highlighted with corresponding color codes. (B) Vector representation of the CHESPA analysis. (C) Fractional activation X and (D) cos *θ* values of MLGM-2013-bound EPAC1 relative to cAMP-bound EPAC1 where values greater than 1 or less than −1 are not within the scale of the plot. (E) Fractional activation X and F) cos *θ* values of I942-bound EPAC1 measured under the same conditions as that of MLGM-2013-bound EPAC1. The asterisks correspond to the residues in the PBC which are more negative in the MLGM-2013-bound structure and the red asterisk marks N275, which exhibits the greatest change. The secondary structure of EPAC1-CNBD is shown in the same way as Fig. 4D.

To test our hypothesis, we measured the EPAC1-CNBD compounded chemical shift changes (ΔCCS) between MLGM-2013 and MLGM-2014 which lacks any phenyl substituents, and therefore, serves as a useful reference ligand to capture the effect of the MLGM-2013 tertiary butyl para substituent (Fig. 5A). Despite the absence of phenyl substituents, MLGM-2014, previously referred to as I178,^5^ was shown to bind EPAC1 and result in an IC_50_ of ∼40 μM.^5^Fig. 5A reports the residue-specific MLGM-2013 *vs.* MLGM-2014 ΔCCS values as well as the corresponding I942 *vs.* MLGM-2014 ΔCCS values as a control. Although the ΔCCS values of the EPAC1 CNBD in the presence of MLGM-2013 or I942 relative to MLGM-2014 are overall similar, the most evident difference is observed in the PBC. MLGM-2013 yields a markedly higher ΔCCS, reflecting additional perturbations in that region due to the bulkier, tertiary butyl moiety at the para phenyl position. These results confirm our hypothesis that the tertiary butyl group of MLGM-2013 interacts with the PBC and that such contacts are unique of MLGM-2013, possibly explaining the enhanced affinity of MLGM-2013 relative to the parent I942 compound.

### The MLGM-2013 mechanism of action

To gain further insight on the possible mechanism of action of MLGM-2013, the CHEmical Shift Projection Analysis (CHESPA)^8,42,43^ was implemented for the MLGM-2013-bound EPAC1 and compared with the CHESPA of I942-bound EPAC1 measured under similar conditions. The CHESPA reports on the ligand-induced shifts in the auto-inhibitory equilibria between inactive and active conformations. It typically requires a reference vector which originates from the inactive protein state (wild type apo in this case) and ends at the active state (cAMP-bound EPAC1 in this case), and a perturbation vector which in this case, is computed from the active state (cAMP-bound EPAC1) and the perturbed state, *i.e.* MLGM-2013-bound EPAC1. The projection of the perturbation vector onto the reference vector yields the cos *θ* values, which indicate the linearity of chemical shifts, and fractional activation (*X*) values, which reflect the extent of ligand-induced protein activation relative to the reference state.^44^ Using the CHESPA vector scheme in Fig. 5B, the fractional activation (*X*) as well as the cos *θ* values were computed for MLGM-2013 (Fig. 5C and D) or I942 (Fig. 5E and F), revealing primarily negative values. This indicates a partial but quite consistent shift towards the apo-inactive conformation of the EPAC1-CNBD, reflecting a partial agonistic activity.

When the CHESPA profiles of MLGM-2013 (Fig. 5C and D) are compared to those of I942 (Fig. 5E and F), one of the most notable differences is observed for PBC residues such as A272, N275 and A277 (asterisks in Fig. 5E). These sites exhibit markedly more negative X values for MLGM-2013 than I942, suggesting a more significant shift towards the inactive state in that region compared to I942. Additionally, MLGM-2013 demonstrates a more negative average X value at the PBC region compared to I942, whereas the average X value for the hinge region is slightly less negative compared to I942 (dotted lines in Fig. 5C and E). These average X values suggest that MLGM-2013-bound EPAC samples an inactive state with PBC out, hinge out with a population of around 60%, while the population of the mixed intermediate with the PBC in, hinge out is negligible. Based on these results, MLGM-2013 promises to serve as a more potent EPAC1-CNBD modulator than I942 with a distinct inhibitory mechanism.

## Discussion and conclusions

We have developed a novel QSAR model for a series of EPAC-specific sulfonamide modulators using a multiple linear regression approach. Our QSAR model resulted in promising correlation coefficients between the actual and predicted affinities for both training and test sets. Hence, the model was used to predict the affinities of a set of new compounds distinct from those used to train or test the model, although sharing a similar scaffold. Based on such predictions, a sulfonamide with a tertiary butyl substituent at the para phenyl position was identified as a promising candidate with a predicted enhanced affinity relative to I942. Fluorescence competition assays confirmed this prediction. Furthermore, NMR analyses revealed that MLGM-2013 shares a similar binding mode as I942, but with more extended contacts with the PBC region of the EPAC1 CNBD due to the tertiary butyl substituent of MLGM-2013. These enhanced contacts lead to inhibitory shifts in the conformational equilibria of the EPAC1 CNBD that suppress the formation of a mixed intermediate previously observed for I942.

The QSAR model proposed here serves as an effective tool to virtually screen compound libraries for EPAC1 binding, thus aiding the identification of novel EPAC1-selective drug candidates. Such tool was not previously available given the absence of prior QSAR studies on EPAC. However, the applicability of such models highly depends on the size of the original datasets utilized to build and train the model. Such datasets should be sufficiently large to generate reliable models and additionally, the molecules should also maintain a conserved structural core for the SPP^21^ to hold. Nevertheless, the protocols and approaches illustrated here can be extended to other ligand databases to develop similar QSAR models for EPAC or other signaling systems, facilitating the design of novel allosteric modulators with enhanced potencies.

## Experimental section

### QSAR model

The I942-based QSAR was developed using the I942 analogues synthesized by Wang *et al.*^19^ The respective molecules were built in MolView,^45^ transferred to the 3D model viewer and the energy of the 3D conformers was minimized using the J mol energy minimization based on the MMFF94 forcefield^46^ and a limit of 100 minimization steps at a time. PaDEL-Descriptor^47^ was used to calculate the 1D and 2D molecular descriptors from the minimized structures. Partition of the molecules into training and test sets was implemented according to an 80 : 20 ratio for the training *vs.* test sets, respectively, and considering their measured affinities, reported as relative fluorescence intensity (RFI) percentages.^19^ Specifically, molecules in each set were chosen to sample the entire spectrum of RFI values. Following these criteria, the original dataset was divided into eleven different training and test partitions. To check for potential outlier RFI values, the Z-score was computed as:^48^1
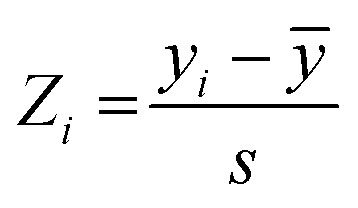
where *y*_*i*_ is the RFI value of a given I942 analog, *ȳ* is the mean and *s* is the standard deviation. Molecules with |*Z*_*i*_| greater than 2.5 are considered outliers.^48^ However, the dataset of I942 analogs did not contain Z-score outliers and therefore, all the molecules were included in the model.

RapidMiner Studio^49^ was used to narrow down the number of descriptors for the QSAR model by applying the forward selection method^50^ on the training set. The method entails sequential addition of molecular descriptors that improve the performance of the model, *i.e.*, descriptors leading to enhanced linear regression correlations. The stopping criteria for the sequential addition are either (1) there is no improvement in model performance or (2) the maximum number of descriptors that satisfy a 5 : 1 ratio for number of molecules *vs.* number of descriptors was reached.^51^ The descriptors chosen were then fed into RapidMiner to generate the linear regression model, which was applied to both the training and test sets to generate a coefficient of multiple determination^31^ (*R*^2^) for each. *R*^2^ is calculated as:2
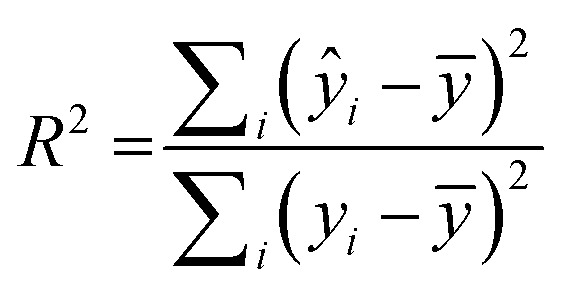
where, *ŷ*_*i*_ is the calculated dependent variable, *i.e.*, the predicted RFI value, *y*_*i*_ is the observed or actual RFI value and *ȳ* is the mean RFI.

An additional parameter reporting on the QSAR quality, known as the root mean squared error (RMSE)^31^ describes the range of error in the model's predictions and is defined as:3
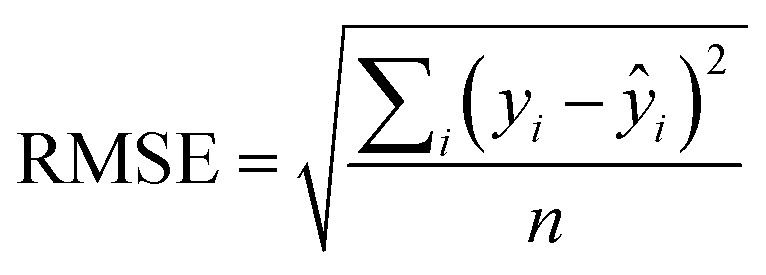
where, *y*_*i*_ is an observed RFI value, *ŷ*_*i*_ is the corresponding predicted RFI value and *n* is the number of molecules in the training set, in this case, 45. The RMSE values of both training and test sets are well below the corresponding standard deviations (*σ*) of the observed RFI percentages, meaning that the predictions are significantly reliable.

As an initial mean of validating the QSAR model, we relied on cross-validation (CV), which is a form of internal validation of the model's predictivity utilizing an approach called the ‘Leave-Many-Out’ (LMO)^52^ method. LMO holds back a portion of the training set as a small test set and applies the model without that test set. The process was repeated for 10 iterations and the squared correlation obtained was represented as an average value of the multiple iterations. The descriptor selection process and QSAR workflow outlined above were repeated for each of the eleven different training and test partitions and average statistical parameters across these partitions were computed.

The Y-randomization coefficient was additionally obtained to validate the robustness of the correlation between the descriptors and the RFI percentages and is described as:4
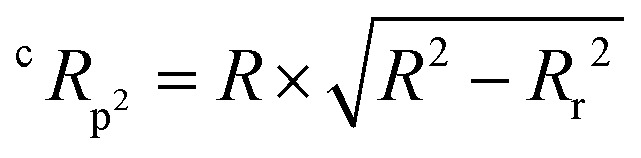
where, *R* is the correlation coefficient of the model before scrambling the *Y* (RFI) values, and *R*_r_ is the mean correlation coefficient of all the permutated models^32^ (1000 iterations in this case).

### Protein purification

The wild-type EPAC1_h_ (149–318) construct was purified according to previously published protocols.^8,14,53–55^ The protein was cultured in either Lysogeny Broth (LB) or ^15^N-labeled M9 minimal media to prepare unlabeled or ^15^N-labeled EPAC1-CNBD, respectively, as needed for fluorescence or NMR measurements.

### Preparation of I942 and MLGM compounds

Compounds were dissolved in deuterated DMSO-*d*_6_ to prepare 10 mM stock solutions. Purity of the compounds was determined to be ≥95% by HPLC. I942 was purchased from Life Chemicals (purity > 99%) and was prepared as a 10 mM stock solution with deuterated DMSO-*d*_6_.

### 8-NBD-cAMP competition assay

8-(2-[7-Nitro-4-benzofurazanyl] aminoethylthio) adenosine-3′,5′-cyclic monophosphate (8-NBD-cAMP) binds to EPAC1-CNBD with high affinity and the binding can be monitored by fluorescence intensity changes.^30^ Unlabeled EPAC1-CNBD was used for this assay. The *K*_D_ measurements of EPAC1-CNBD in complex with either I942, MLGM-2013, or MLGM-2017 were recorded from the decrease in fluorescence intensity as a result of 8-NBD-cAMP competitive displacement.^8,30^ The compounds were added at concentrations between 0 to 300 μM to solutions of 2.5 μM and 0.5 μM of EPAC1-CNBD and 8-NBD-cAMP, respectively. The NMR buffer (*vide infra*) was used to bring the final volume of the samples to 250 μL. Samples were added to Corning 96-well half area plates (120 μL per well) after an incubation period of at least 30 minutes at room temperature to allow for equilibration. A Cytation 5 plate reader was used to scan the plate using excitation and emission wavelengths of 485 nm and 535 nm, respectively. The equation used for fitting the competitive binding isotherms was applied as previously described.^56^ The resulting RFI values provide an estimation of experimental relative affinity rankings, which were compared to the rankings predicted through QSAR.^57^

### NMR measurements

NMR experiments were acquired using a Bruker Avance or NEO 700 MHz spectrometer with a TCI cryoprobe. For the HSQC experiments, 350 μM of the ligand (I942/MLGM) was added to 50 μM of EPAC1-CNBD in NMR buffer with 5% D_2_O. The same volume of DMSO-*d*_6_, present in the NMR samples with ligands, was added to the apo sample to exclude the effect of DMSO-*d*_6_ from the chemical shift perturbation assessment. The ^15^N–^1^H-HSQC experiment utilized an Echo and Anti-echo PFG selection along with a water flip-back and the operating temperature was 306 K. The time domain digitization points were 2048 and 128 for the ^1^H and ^15^N dimensions, respectively, and the spectral widths were 16.23 ppm for ^1^H and 38 ppm for ^15^N. The number of scans was 64 and the recycle delay was 1 second. The spectra were processed in TopSpin (Bruker), where the size of the real spectrum (SI) was 2048 and 512 for the ^1^H and the ^15^N dimensions, respectively. Sine bell shift (SSB) values of 2 and 3 were applied for the ^1^H and ^15^N dimensions, respectively, and a sine squared window function (WDW) was applied for both dimensions. Forward line prediction (LPfc) was utilized for the ^15^N dimension, where the number of LP coefficients was 32. The chemical shifts were referenced to ^15^N-acetyl glycine and were assigned through comparison with the apo and cAMP-bound EPAC1-CNBD at 306 K that were previously acquired and assigned.^8^

The compounded chemical shift differences (ΔCCS) between ligand bound EPAC1 and the apo form were calculated using the following equation:5
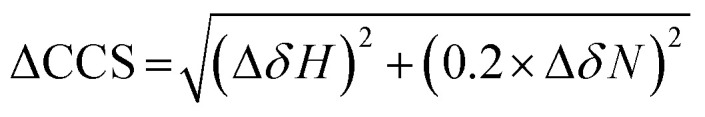


Samples for 1D saturation transfer difference (STD) were prepared using a 50 μM EPAC1-CNBD solution that was buffer-exchanged with a 20 mM sodium phosphate buffer containing 50 mM NaCl, pH 7.4 and 99.9% D_2_O. PD-10 Desalting columns (GE Healthcare) were used to facilitate the exchange by a gravity protocol. 350 μM of MLGM-2013 (final concentration) was added to 50 μM of EPAC1-CNBD and the saturation frequency in the STD experiments was set to 0.8 ppm to saturate the region of protein peaks (*i.e.*, methyl region) that is further away from the MLGM-2013's signal. An off-resonance saturation of 30 ppm was applied to the STR experiments and the STD/STR ratios normalized to the largest value were compared to those acquired for I942.^8^ The spectra were referenced to DMSO (2.48 ppm) and the assignments of MLGM-2013 were obtained by comparison with previously established assignments for I942.^8^ The STD spectra were acquired at 298 K with a time domain of 32768 points and number of scans of 128 and 1024 for STR and STD experiments, respectively. Eight dummy scans were used for both STD and STR. The spectral width was 11.7057 ppm, and the transmitter frequency was set to 4.697 ppm.

The chemical shift projection analysis (CHESPA) was implemented according to previous protocols^8,42,43^ and using the NMRFAM-SPARKY plugin.^44^ The reference vector (*A*) is defined from the apo to the cAMP-bound EPAC1 state, while the perturbation vector (*B*) is defined from the cAMP-bound form to the I942-analog ligand-bound form. The minimum cut-off for the ΔCCS values of both vectors was set to 0.02 ppm and the cos *θ* and fractional activation (*X*) values were computed according to the following formulae:6
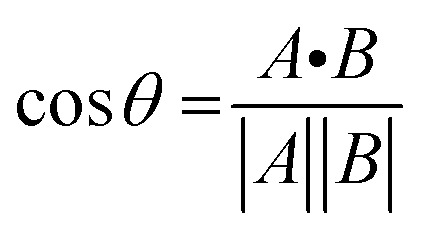
7
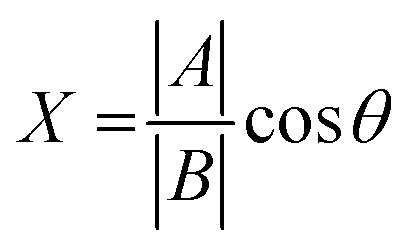


## Author contributions

The manuscript was written by H. M. and G. M. through the contributions of all authors. All authors have given approval to the final version of the manuscript.

## Abbreviations

cAMP: Cyclic adenosine monophosphate
EPAC: Exchange protein activated by cAMP
GEF: Guanine nucleotide exchange factor
GDP: Guanosine diphosphate
GTP: Guanosine triphosphate
CNBD: Cyclic nucleotide binding domain
PBC: Phosphate binding cassette
NOE: Nuclear Overhauser effect
BBR: Base binding region
QSAR: Quantitative structure activity relationship
SPP: Similarity-property principle
RFI: Relative fluorescence intensity
8-NBD-cAMP: 8-(2-[7-Nitro-4-benzofurazanyl] aminoethylthio) adenosine-3′,5′-cyclic monophosphate
K_D_: Dissociation constant
NMR: Nuclear magnetic resonance
HSQC: Heteronuclear single quantum coherence
CCS: Compounded chemical shift
STD: Saturation transfer difference
STR: Saturation transfer reference
CHESPA: Chemical shift projection analysis
CHESCA: Chemical shift covariance analysis

## Conflicts of interest

The authors declare no competing financial interest.

## Supplementary Material

CB-003-D2CB00106C-s001
