# Supplementary material for: QSAR models reveal new EPAC-selective allosteric modulators
Source: RSC Chem Biol. 2022 Aug 3;3(10):1230–9. doi: 10.1039/d2cb00106c (PMC9533425; doi:10.1039/d2cb00106c)
Supplement: CB-003-D2CB00106C-s001 [file CB-003-D2CB00106C-s001.pdf]

Electronic Supplementary Material (ESI) for RSC Chemical Biology.
This journal is © The Royal Society of Chemistry 2022

Supplementary Material for

QSAR Models Reveal New EPAC-Selective Allosteric Modulators

Hebatallah Mohamed[1], Hongzhao Shao[1], Madoka Akimoto[1], Patrick Darveau[1], Marc R. MacKinnon[2], Jakob Magolan[2], Giuseppe Melacini[1,2*]

[1]Department of Chemistry and Chemical Biology, McMaster University, Hamilton ON, L8S 4L8, Canada.
[2]Department of Biochemistry and Biomedical Sciences, McMaster University, Hamilton ON, L8S 4L8, Canada.
[*]**Corresponding author email - melacin@mcmaster.ca**

Contents: supplemental Tables S1-S2, supplemental Figures S1-S4 and supplemental compound synthesis and purity section (Figure S5-S11)

Supplemental Tables

Table S1: Code names for the test set molecules used for each of the 10 dataset partitions. The training set molecules are the remaining part of the dataset (~ 80%).

Division 1	Division 2	Division 3	Division 4	Division 5	Division 6	Division 7	Division 8	Division 9	Division 10
25ad	12e	12g	12g	12d	25ac	12a	25ad	25aa	12c
25b	12f	25aa	25a	25ac	25c	25i	25e	25b	12e
25e	25ab	25g	25ab	25c	25i	25k	25j	25m	12f
25h	25e	25h	25d	25v	25l	25u	25o	25v	25g
25p	25j	25i	25q	25w	25m	25w	25p	25z	25o
25r	25l	25q	25y	25x	25z	25x	25r	9b	25r
25t	25m	25s	25z	9f	9e	25z	25v	9e	25w
9b	25n	25y	9i	9h	9l	9g	25y	9g	25x
9e	25r	9o	9k	9j	9q	9k	9b	9i	9j
9j	9l	9r	9p	9n	9r	9m	9c	9m	9n
9n	9m	9s	9q	9p	9s	9q	9d	9o	9s

Table S2: Parameters for the QSAR model developed for the I942 analogues* with a non-zero intercept

	Training Set	Test Set	Cross-Validation	Threshold
R^2	0.808 ± 0.057	0.577 ± 0.167	0.772 ± 0.055	$R^2 > 0.600$
σ	27.69 ± 0.60	28.69 ± 2.40	-	-
RMSE	11.83 ± 1.81	19.09 ± 3.13	12.78 ± 1.50	RMSE < σ
k	0.808 ± 0.057	0.628 ± 0.197	-	$0.850 \leq k \leq 1.150$

* *Standard deviations were computed using data from eleven different partitioning of training vs. test sets.*

Supplemental Figures

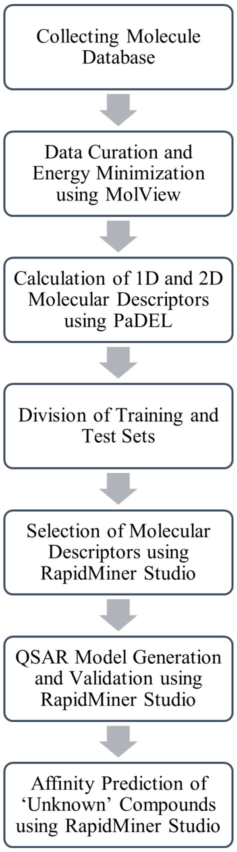

Figure S1: Flowchart describing the workflow of the QSAR model generation and validation

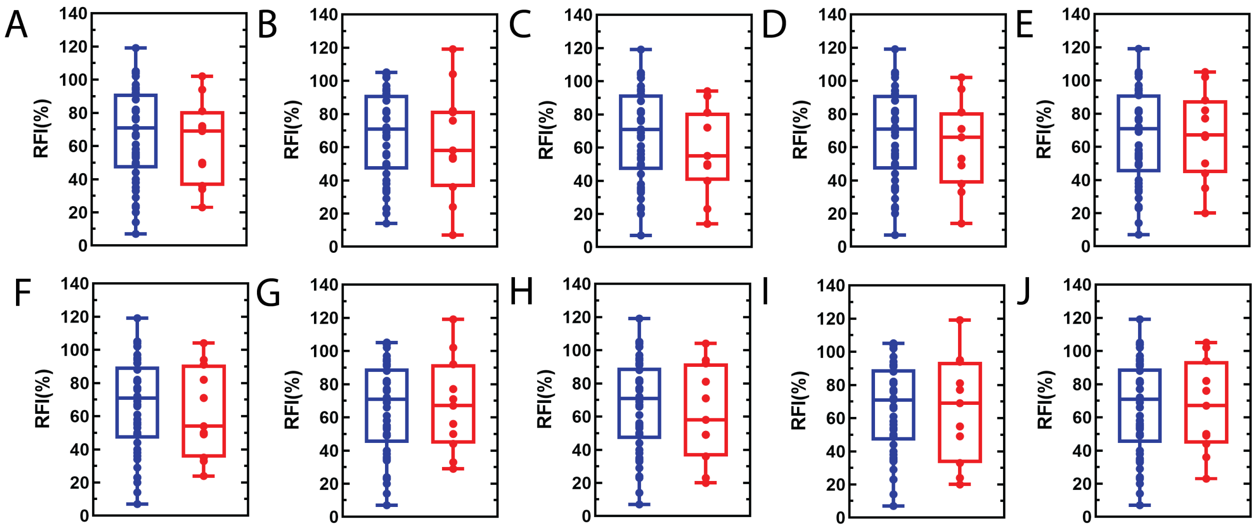

Figure S2: (A-J) Box plot representations for the range of RFI values of different training (blue) and test (red) set divisions. The number of training set molecules is 45 and test set molecules is 11 for all the divisions.

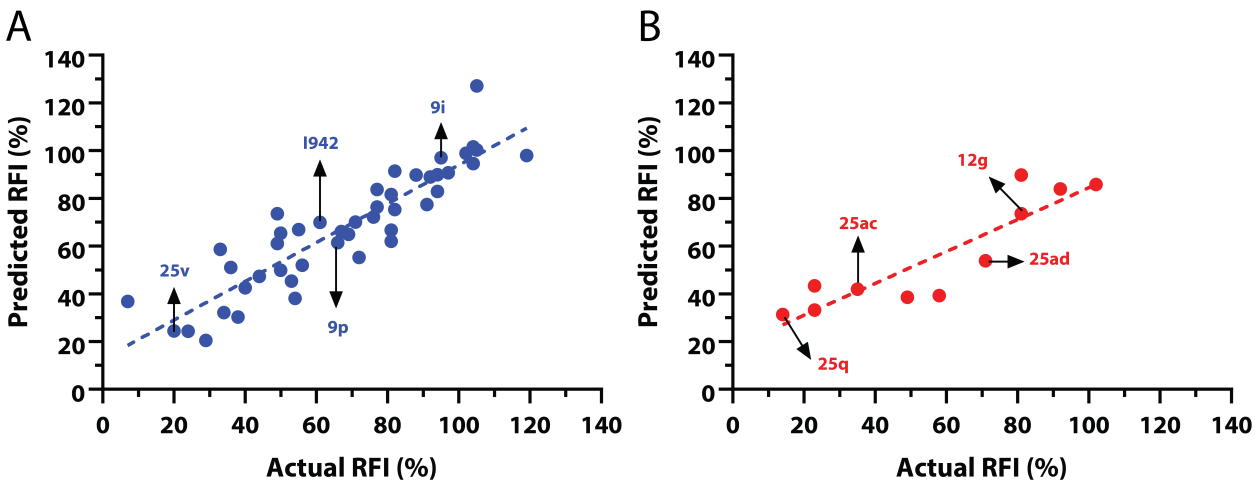

Figure S3: Correlation plots for the A) training and B) test sets of the I942 analogues with a non-zero intercept.

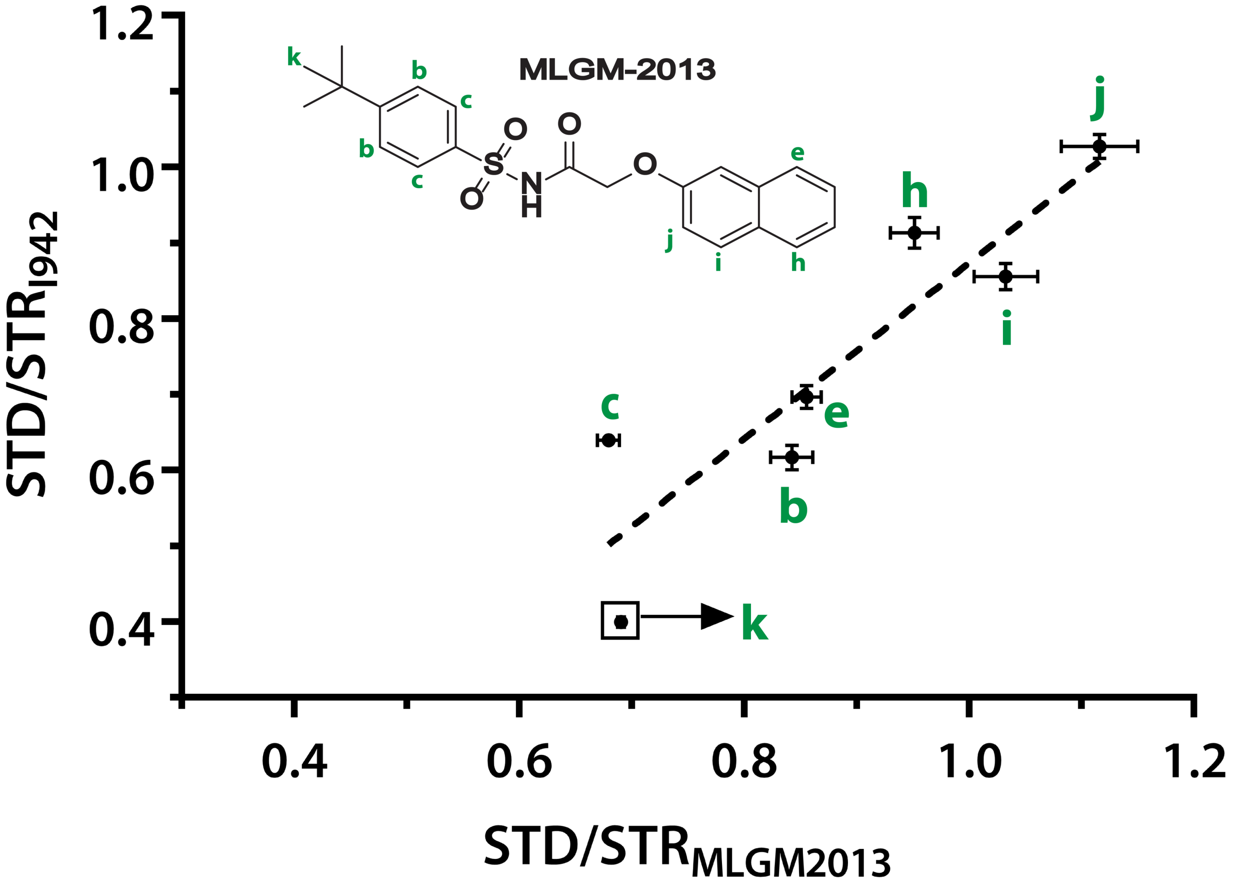

Figure S4: Correlation plot for the STD/STR intensity ratios of protons of MLGM-2013 against protons of I942. The point marked by a box and labeled as 'k' corresponds to the protons that are different between MGLM-2013 (*tert*-butyl) and I942 (methyl) and the error bars for that point are within the symbol size.

Equation of the model used to predict the affinities of the MLGM compounds:

Equation (S1):

$$RFI = 24.2 * AATS5s + 10.8 * ATSC8e + 410.7 * AATSC3p + 39.0 * GATS5s + 178.5 * VC\text{-}5 - 1.1 * minsOm + 0.1 * maxHBint4 - 87.0$$

Synthetic Chemistry Experimental Procedures

General: ^{1}H NMR spectra were acquired at 400 MHz. ^{1}H NMR and ^{13}C NMR chemical shifts are reported in parts per million (ppm) relative to tetramethylsilane (TMS), with calibration of the residual chloroform solvent as follows: δ_H 7.26, δ_C 77.16).

4-(tert-butyl)benzene sulfonamide and 2,4-dimethylbenzenesulfonamide were purchased from Oakwood Chemical. All other reagents and solvents were purchased from Sigma Aldrich or Fisher Scientific. 2-((2-oxo-2H-chromen-7-yl)oxy)acetic acid, 2-(naphthalen-2-yloxy)acetic acid, 2-(naphthalen-2-yloxy)-N-(phenylsulfonyl)acetamide (MLGM2014) were synthesized according to previously established protocols.[1,2]

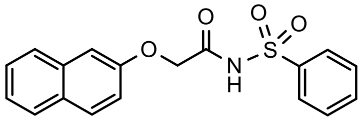

MLGM2014
2-(naphthalen-2-yloxy)-*N*-(phenylsulfonyl)acetamide

Compounds **MLGM2013**, **MLGM2014**, and **MLGM2017** were confirmed to be >95% pure by HPLC (*see HPLC traces below*).

Synthesis of MLGM-2013:

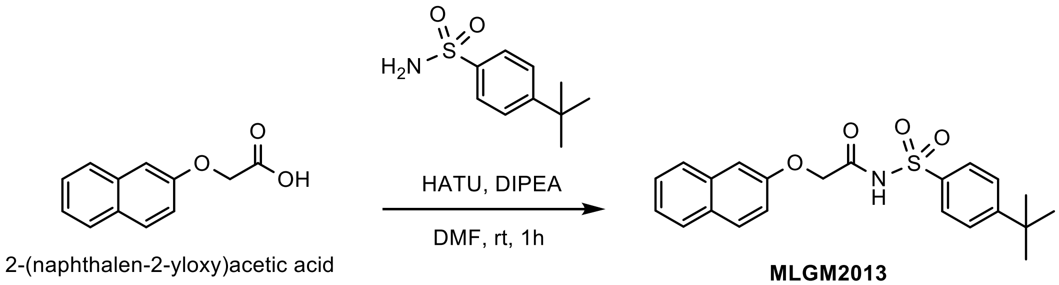

2-(naphthalen-2-yloxy)acetic acid **MLGM2013**

In a round-bottom flask, 68.6 mg (0.34 mmol, 1 equiv.) of **2-(naphthalen-2-yloxy)acetic acid**, 144.7 mg (0.68 mmol, 2 equiv.) of **4-(*tert*-butyl)benzene sulfonamide** and 0.25 mL (1.36 mmol, 4 equiv.) of **DIPEA** were loaded in 2 mL (0.2 M) of **DMF**. To a stirring solution, 154.9 mg (0.41 mmol, 1.2 equiv.) of **HATU** was added portion wise. The resulting mixture was stirred at room temperature for 1 hour. It was then diluted with EtOAc and washed with «half-brine» (8x), dried over $MgSO_4$, filtered and concentrated under reduced pressure. Purification was done by reverse phase chromatography, loaded in DMSO onto a C18 Silicycle column, eluted from 5 to 100% CH_3CN/H_2O (with 0.1% formic acid) over 15 column volumes (CVs). Fractions rich in desired product by LRMS were combined and concentrated under reduced pressure, then taken up into nanopure water, frozen and lyophilized to afford 20.1 mg (15% yield) of **MLGM-2013** as a light-brown solid.

^{1}H NMR (400 MHz, Chloroform-*d*) δ 8.94 (s, 1H), 8.02 – 7.94 (m, 2H), 7.85 – 7.70 (m, 2H), 7.70 – 7.63 (m, 1H), 7.57 – 7.49 (m, 2H), 7.47 (ddd, *J* = 8.3, 6.9, 1.4 Hz, 1H), 7.41 (ddd, *J* = 8.1, 6.8, 1.3 Hz, 1H), 7.18 (dd, *J* = 9.0, 2.6 Hz, 1H), 7.02 (d, *J* = 2.6 Hz, 1H), 4.59 (s, 2H), 1.35 (s, 9H).

^{13}C NMR (101 MHz, Chloroform-*d*) δ 166.02, 154.24, 134.98, 134.04, 130.32, 129.77, 128.42, 127.78, 127.03, 126.97, 126.12, 124.79, 117.82, 107.70, 67.24, 35.36, 31.03.

Synthesis of MLGM-2017:

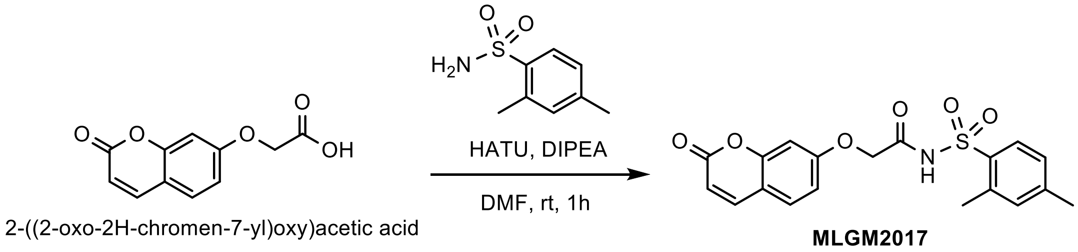

In a round-bottom flask, 100 mg (0.46 mmol, 1 equiv.) of **2-((2-oxo-2H-chromen-7-yl)oxy)acetic acid**, 168.2 mg (0.91 mmol, 2 equiv.) of **2,4-dimethylbenzenesulfonamide** and 0.3 mL (1.82 mmol, 4 equiv.) of **DIPEA** were loaded in 2 mL (0.2 M) of **DMF**. To a stirring solution, 154.9 mg (0.41 mmol, 1.2 equiv.) of **HATU** was added portion wise. The resulting mixture was stirred at room temperature for 1 hour. It was then diluted with EtOAc and washed with «half-brine» (8x), dried over $MgSO_4$, filtered and concentrated under reduced pressure. Purification was done by reverse phase chromatography, loaded in DMSO onto a C18 Silicycle column, eluted from 5 to 100% CH_3CN/H_2O (with 0.1% formic acid) over 15 column volumes (CVs). Fractions rich in desired product by LRMS were combined and concentrated under reduced pressure, then taken up into nanopure water, frozen and lyophilized to afford 88.1 mg (47% yield) of **MLGM-2017** as a beige solid.

^{1}H NMR (400 MHz, Chloroform-*d*) δ 8.86 (s, 1H), 8.08 (d, *J* = 8.1 Hz, 1H), 7.66 (d, *J* = 9.5 Hz, 1H), 7.45 (d, *J* = 8.6 Hz, 1H), 7.24 – 7.17 (m, 1H), 7.11 (d, *J* = 1.8 Hz, 1H), 6.88 (dd, *J* = 8.6, 2.5 Hz, 1H), 6.77 (d, *J* = 2.5 Hz, 1H), 6.34 (d, *J* = 9.5 Hz, 1H), 4.53 (s, 2H), 2.56 (s, 3H), 2.40 (s, 3H).
^{13}C NMR (101 MHz, Chloroform-*d*) δ 165.02, 160.56, 159.22, 155.78, 145.79, 143.00, 137.83, 133.46, 133.20, 131.90, 129.55, 127.35, 114.89, 114.35, 112.45, 102.44, 67.46, 21.62, 20.49.

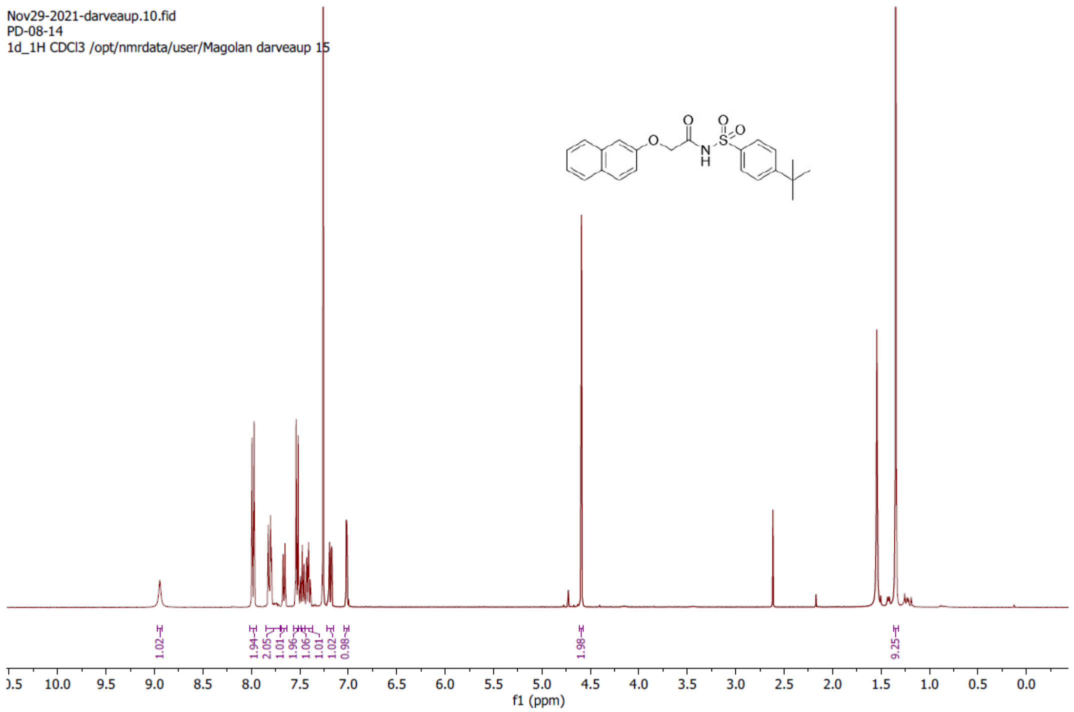

Figure S5: MLGM-2013 ^{1}H NMR (400 MHz, $CDCl_3$-*d*)

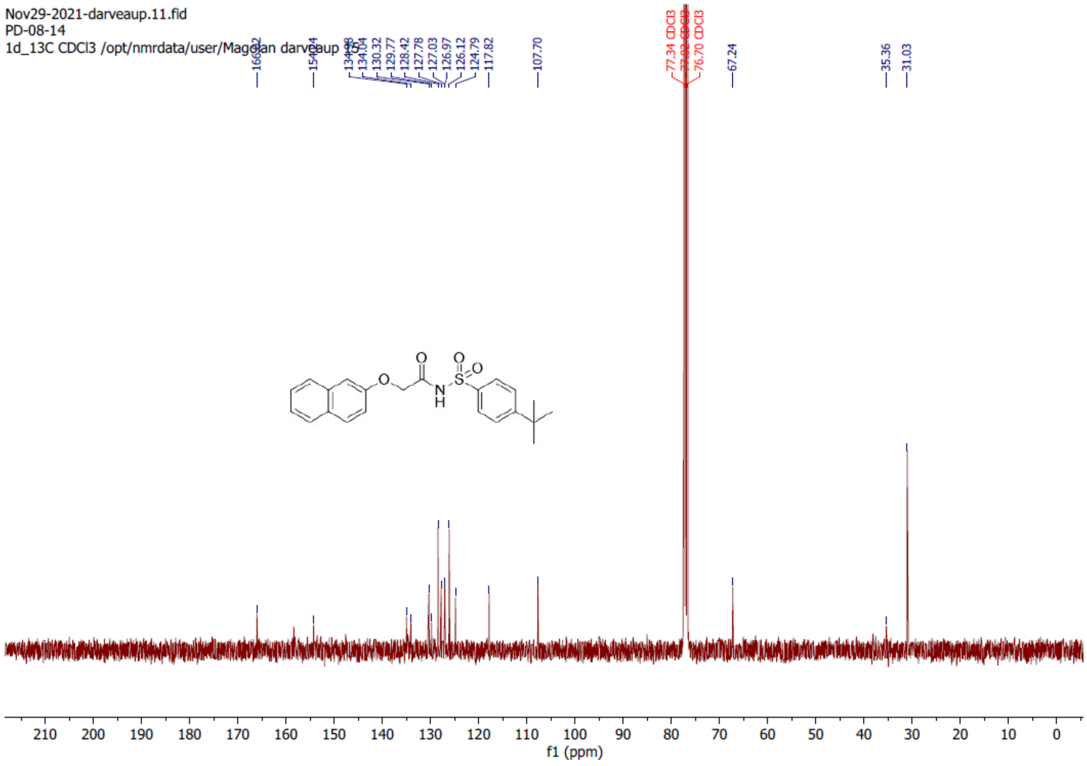

Figure S6: MLGM-2013 ^{13}C NMR (101 MHz, $CDCl_3$-*d*)

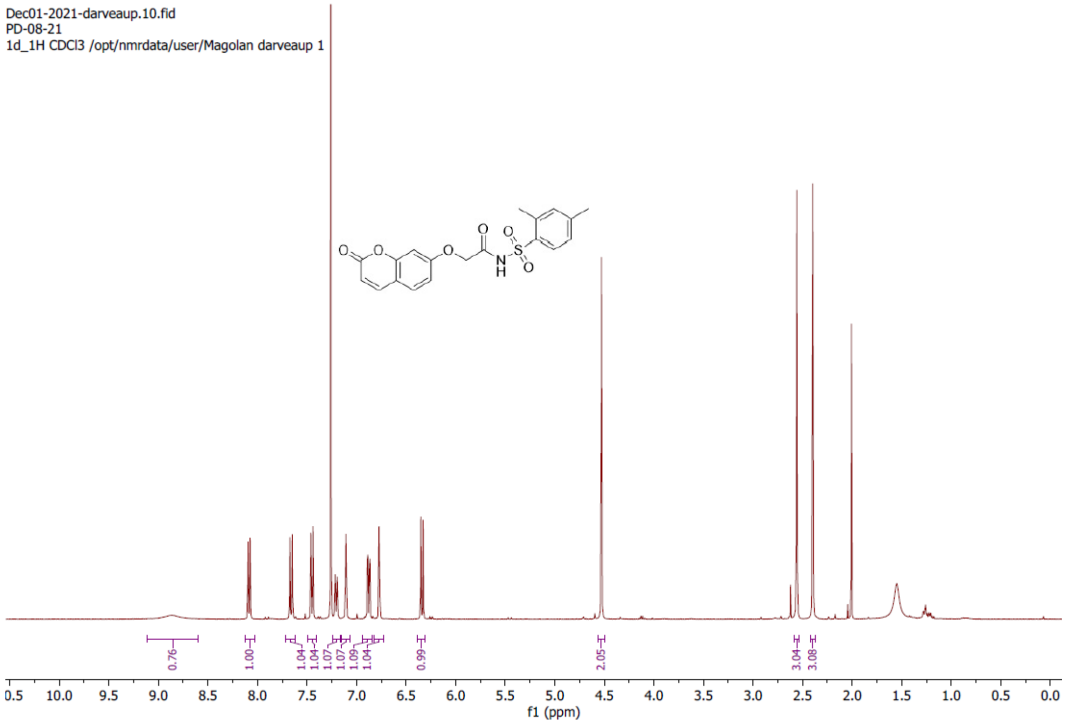

Figure S7: MLGM-2017 ^{1}H NMR (400 MHz, $CDCl_3$-*d*)

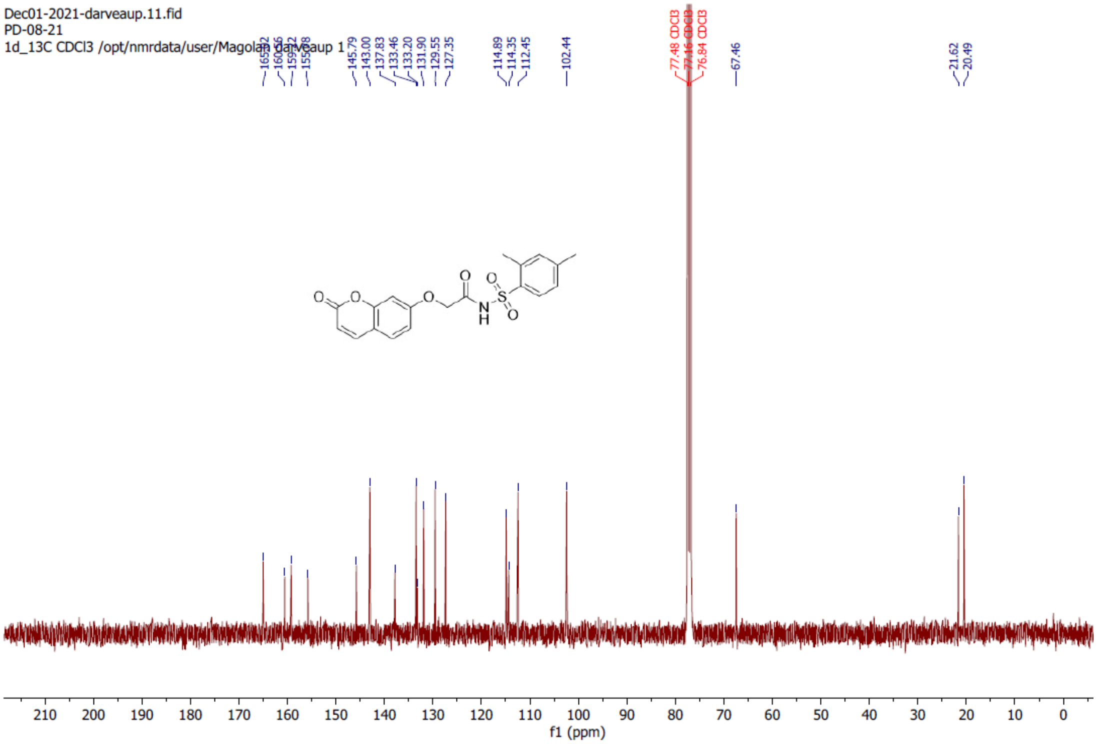

Figure S8: MLGM-2017 ^{13}C NMR (101 MHz, $CDCl_3$-*d*)

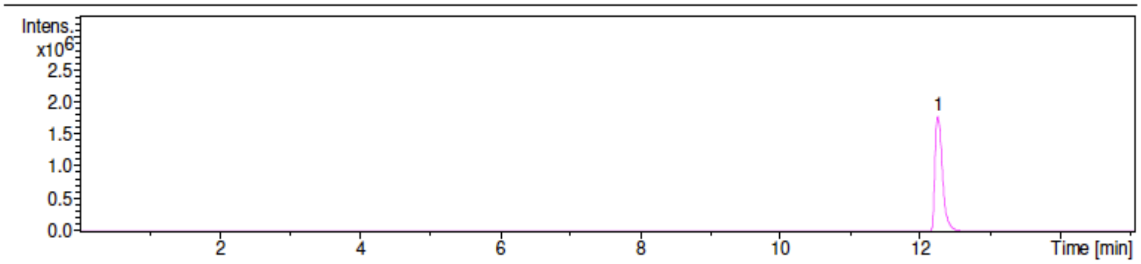

#	RT [min]	Area	Area Frac. %
1	12.2	13322497	100.0

Figure S9: HPLC trace of compound **MLGM-2013**

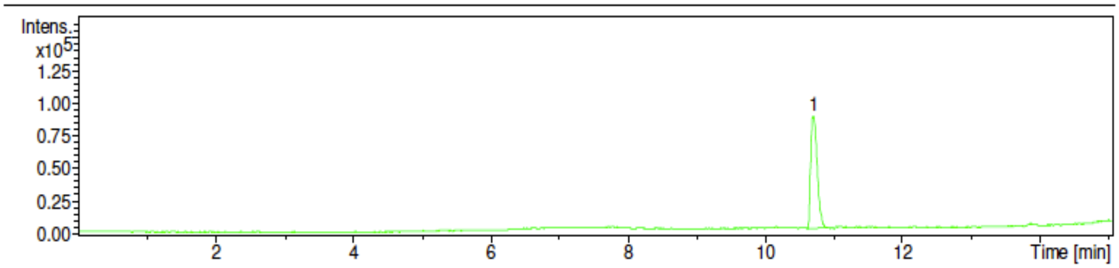

#	RT [min]	Area	Area Frac. %
1	10.7	559355	100.0

Figure S10: HPLC trace of compound **MLGM-2014**

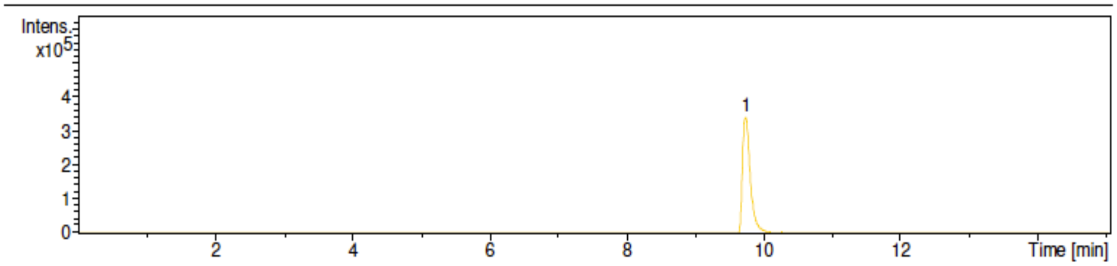

#	RT [min]	Area	Area Frac. %
1	9.7	2697892	100.0

Figure S11: HPLC trace of compound **MLGM-2017**

References:

(1) Peperidou, A.; Silvia, B.; Bozdag, M.; Hadjipavlou-Litina, D.; Supuran, C.T. Novel 6- and 7-Substituted Coumarins with Inhibitory Action against Lipoxygenase and Tumor-Associated Carbonic Anhydrase IX. *Molecules* **2018**, *23* (*1*), 153.

(2) Wang, P.; Luchowska-Stańska, U.; van Basten, B.; Chen, H.; Liu, Z.; Wiejak, J.; Whelan, P.; Morgan, D.; Lochhead, E.; Barker, G.; Rehmann, H.; Yarwood, S. J.; Zhou J. Synthesis and Biochemical Evaluation of Noncyclic Nucleotide Exchange Proteins Directly Activated by cAMP 1 (EPAC1) Regulators. *J. Med. Chem.* **2020**, 63(*10*), 5159.
